# Long-term outcomes after particle radiation therapy in patients with nasopharyngeal adenoid cystic carcinoma

**DOI:** 10.1186/s12885-024-12471-8

**Published:** 2024-06-18

**Authors:** Weixu Hu, Jiyi Hu, Qingting Huang, Jing Gao, Haojiong Zhang, Lin Kong

**Affiliations:** 1 Department of Radiation Oncology, Shanghai Proton and Heavy Ion Center, Fudan University Cancer Hospital, Shanghai, 201315 China; 2 Shanghai Key Laboratory of radiation oncology (20dz2261000), Shanghai, 201315 China; 3 Shanghai Engineering Research Center of Proton and Heavy Ion Radiation Therapy, Shanghai, 201315 China

**Keywords:** Nasopharyngeal adenoid cystic carcinoma (NACC), Proton therapy, Carbon ion radiotherapy (CIRT), Long-term outcomes

## Abstract

**Background:**

Nasopharyngeal adenoid cystic carcinoma (NACC) is a relatively rare salivary gland tumor that is generally associated with poor outcomes. High-dose radiotherapy is a key treatment for patients with NACC. This study reported the long-term efficacy and safety of particle beam radiation therapy (PBRT) for NACC.

**Methods and materials:**

Twenty-six patients with nonmetastatic NACC who received definitive PBRT alone were included in this retrospective study. The majority of patients (92.3%) had locally advanced disease. Twenty-five (96.15%) patients received intensity-modulated proton radiotherapy (IMPT) followed by a carbon ion radiotherapy (CIRT) boost, and one patient received CIRT alone. Overall survival (OS), local control (LC), regional control (RC), and distant metastasis control (DMC) rates were calculated via the Kaplan-Meier method.

**Results:**

The median follow-up time was 46.95 months for the entire cohort. Seven patients experienced local recurrence, and one patient experience neck lymph node recurrence. The 3- and 4-year OS, LC, RC, and DMC rates were 100% and 91.7%, 92.3% and 84.6%, 95.8% and 87.8%, and 90.2% and 71.3%, respectively. A total of 91.3% of the patients achieved complete remission of gross tumors at 1 year after PBRT. Severe acute toxicity was observed in only two patients. A grade 4 decrease in visual acuity was observed in one patient with orbital apex invasion. No late grade 3 or 5 toxicity was observed.

**Conclusion:**

Definitive PBRT provided a satisfactory 4-year OS for patients with locally advanced NACC. The toxicity was acceptable and mild. Further follow-up is necessary to confirm the efficacy and safety of definitive PBRT for patients with NACC.

## Introduction

Adenoid cystic carcinoma (ACC) is a relatively rare salivary gland tumor that accounts for approximately 1% of all head and neck malignancies and 25–30% of all malignant salivary gland tumors. Nasopharyngeal ACC (NACC) is a distinct subgroup of ACC that accounts for fewer than 5% of head and neck ACC cases and is associated with a worse outcome [1–3].

Because of its radio-resistant nature and limited accessibility during surgery due to its anatomical complexity, NACC is difficult to treat. Consequently, high-dose radiotherapy (RT) has become the main approach for managing these tumors. However, the tolerance of critical anatomical areas such as the brainstem and the temporal lobes surrounding the nasopharynx is relatively poor. Thus, a careful balance needs to be maintained between disease control and treatment-related toxicity. The outcomes of photon-based definitive RT over the past decade have been suboptimal, with 5-year local control (LC) rates ranging between 30% and 56% and 5-year overall survival (OS) rates ranging between 55% and 61% (4.5). Moreover, a recent retrospective national population-based study reported an OS rate as low as 40% for patients with advanced-stage disease [4].

Proton beams and carbon ion beams have Bragg peaks, and these unique physical characteristics allow for high dose coverage to the target region with a relatively low dose to the surrounding normal tissues [5]. In addition to their advantage of dose distribution, carbon ion beams have higher linear energy transfer (LET) and relative biological effectiveness (RBE) than photon or proton beams [6–9]. We previously reported the short-term outcomes of patients with NACC who were treated with particle radiation therapy (PBRT), with 2-year OS, LC, and DMC rates of 100%, 94.4%, and 84.8%, respectively [10]. In the present study, we examined the long-term outcomes of 26 patients with NACC who were treated with PBRT alone.

## Methods and materials

### Patients and pretreatment evaluations

Between July 2016 and November 2021, the data of 26 consecutive patients with nonmetastatic NACC were summarized and included in this retrospective study. Except for one patient who experienced recurrence, the remaining patients had newly diagnosed disease that was confirmed by pathology. All patients were treated with PBRT (carbon ion RT [CIRT] or a combination of CIRT and intensity-modulated proton RT [IMPT]) alone.

Evaluations before PBRT included a complete medical history and physical examination, nasopharyngoscopy, complete blood count (CBC), serum electrolytes, electrocardiography, urinalysis, and magnetic resonance imaging (MRI) of the nasopharynx and bilateral neck. Chest computed tomography (CT), abdominal ultrasonography, and bone scans were performed to evaluate the status of distant metastasis. Whole-body positron emission tomography (PET) was performed if clinically indicated. The eighth edition of the American Joint Committee on Cancer (AJCC) staging system was used for all patients.

### Particle beam radiation therapy

Individualized dual-component polyurethane foam (Alpha Cradle, Smithers Medical Product, North Canton, OH, USA) and thermoplastic masks were used for all patients in the supine position. CT for simulation of the head and neck region with a 1.5-mm slice thickness without IV contrast was performed for all patients. MRI–CT fusion imaging was performed for target contouring for all patients. The gross tumor volume of the nasopharynx and positive retropharyngeal lymph nodes (GTV-np) was defined as the gross tumor identified on imaging studies. The gross tumor volume of positive neck lymph nodes (GTV-n) was defined as the gross tumor identified on clinical examination or imaging studies. A 1- to 3-mm margin (depending on the critical organs at risk [OARs]) was added to the GTVs to create the clinical target volume (CTV)-G. CTV-1 included the entire nasopharynx, skull base, and bilateral level II and level Va nodal region for N0 or partial N1 (only positive retropharyngeal lymph nodes) patients. CTV-2 included the ipsilateral level III, level IV and level Vb-c nodal regions for N1 patients with positive unilateral neck lymph nodes. For patients with N2 disease, the bilateral level III, level IV and level Vb-c nodal regions were included in CTV-2. An additional 3 to 6 mm margin was added to the CTVs to create the planning target volume (PTV) for range uncertainty and setup error.

The dose of PBRT was measured using gray equivalents (GyEs). The dose constraints of the OARs were based on the normal tissue tolerance described by Emami et al. [11]. The dose limits for the optic nerve and temporal lobes were based on the guidelines of the National Institute of Radiation Science (NIRS) of Japan [12]. IMPT or CIRT was delivered using pencil beam scanning (PBS) on the IONTRIS system (Siemens Healthineers, Erlangen, Germany). Two or three fields were typically delivered using multifield optimization (MFO). Weekly CT scans without IV contrast were required to assess any anatomic changes.

### Follow-up and toxicity evaluation

Daily physical examination was completed during treatment for all patients. The first follow-up after PBRT was performed within 4–6 weeks. Subsequently, all patients were encouraged to attend follow-up every 3 months in the first 2 years, every 6 months in the following 3 years, and annually after 5 years. MRI of the head and neck region was performed at each follow-up. Every 6 to 12 months, chest CT, abdominal ultrasonography, and bone ECT were conducted.

### Statistical analysis

The duration of OS was defined as the time from the diagnosis of the primary disease until death or the last follow-up. The times to local recurrence, regional failure, and distant metastasis were measured from the initial diagnosis to the corresponding failure. Survival rates were calculated using the Kaplan‒Meier method. The tumor response after treatment was measured according to the Response Evaluation Criteria in Solid Tumors (RECIST) version 1.1. Acute toxicities were defined as adverse events that occurred within 90 days after the initiation of PBRT. Late toxicities were defined as those that occurred after 3 months or adverse events that persisted for more than 3 months after completion of treatment. All analyses were performed using the statistical program SPSS (version 26.0).

## Results

### Patient characteristics and treatment

A total of 28 consecutive patients with nonmetastatic NACC were treated with particle RT alone at the Shanghai Proton and Heavy Ion Center between July 2016 and November 2021. Of these patients, 2 patients were excluded from this analysis due to reirradiation; 24 (92.3%) patients had locally advanced disease (T3 and T4), with patients with T4 disease accounting for 80.77% of the entire group. Orbit or orbital apex invasion was present in five patients (19.23%), and 42.31% of patients (*n* = 11) underwent transnasal route surgery before particle RT, all of whom had residual disease. The median tumor volume was 47.75 ml (9.1–145.31 ml). Table 1 summarizes the patients’ baseline and treatment characteristics.

**Table 1:** Baseline and treatment characteristics

Characteristics	No. of patients (%)
**Age**	
Median (range) – year	45.5 (21–71)
≤50-year	15 (57.69%)
>50-year	11 (42.31%)
**Gender**	
Female	19 (73.08%)
Male	7 (26.92%)
**Tumor category**	
T1	1 (3.85%)
T2	1 (3.85%)
T3	3 (11.54%)
T4	21 (80.77%)
**Node category**	
N0	15 (57.69%)
N1	8 (30.77%)
N2	3 (11.54%)
**Disease stage**	
I	1 (3.85%)
II	1 (3.85%)
III	3 (11.54%)
IV	21 (80.77%)
**Orbit/Orbital apex invasion**	
Yes	5 (19.23%)
No	21 (80.77%)
**Disease status**	
Newly diagnosed	25 (96.15%)
Recurrent	1 (3.85%)
**Surgery** ^†^	
Yes	11 (42.31%)
No	15 (57.69%)
**Gross tumor**	
With gross tumor	26 (100%)
Without gross tumor	0
Median tumor volume – mL	47.75 (9.1-145.31)
**Radiotherapy modality**	
IMPT + CIRT	25 (96.15%)
CIRT alone^‡^	1 (3.85%)
Median dose to GTV - GyE	73.5 (70-73.5)

### Particle RT

Twenty-five patients (96.15%) received proton and carbon ion mixed-beam intensity-modulated RT. CTV-1 (including the entire nasopharynx, skull base, bilateral level II and level Va nodal regions) and CTV-2 (including the level III, level IV and level Vb-c nodal regions for N + patients) were treated with proton therapy to 50.4–56 GyE in 28 fractions, followed by a CIRT boost to 15–17.5 GyE in five fractions to GTVs (including tumors in the head and positive lymph nodes). One patient with recurrent disease after surgical treatment received CIRT therapy alone (70 GyE in 20 fractions) to the recurrent tumor and 60 GyE in 20 fractions to the high-risk region but not to the neck. Bilateral upper neck irradiation was performed for N0 patients except for those with recurrent disease.

### Treatment response and survival

**Fig. 1:**
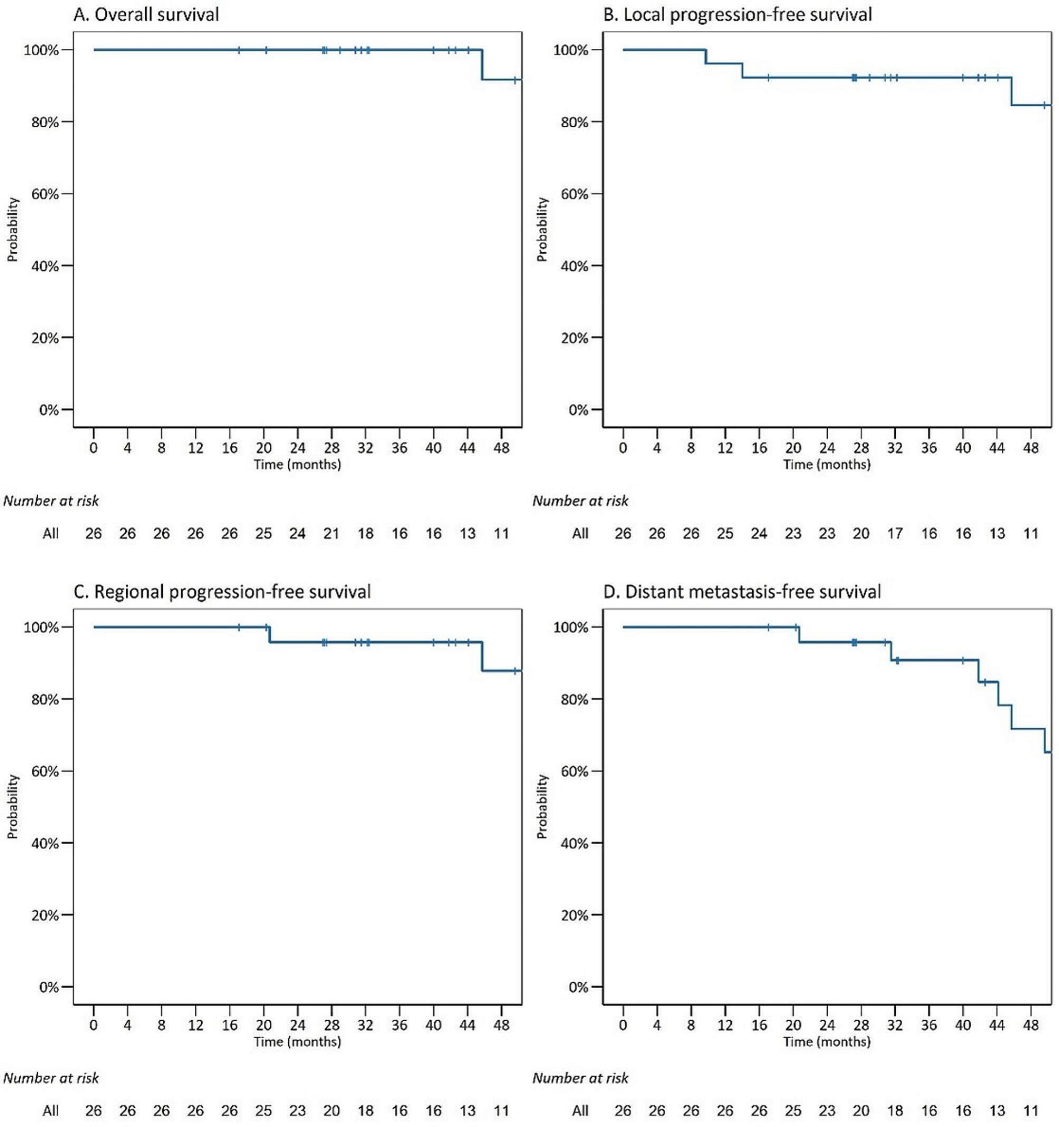
Survival curves of the OS, LC, RC, and DMC rates for the study cohort. OS, overall survival; LC, local control; RC, regional control; DMC, distant metastasis control

The median follow-up for the entire cohort was 46.95 (range 17.4–77.6) months, all patients were alive at 3 years (95% CI 100–100%), and the 4-year OS was 91.7% (95% CI 77.3–100%) (Fig. 1A). Seven patients experienced local recurrence, 2 of whom had distant metastasis. Four local recurrence events occurred within the GTV, and 2 occurred at the margin of CTV1. Neck lymph node relapse occurred in one patient who had N1 disease before particle RT. However, none of the patients with stage I–II disease experienced local or regional failure. A total of ten patients developed distant metastasis, and the most common site was the lung (7 patients), followed by the liver (1 patient) and bone (1 patient). One patient had both lung and liver metastasis. Of the 23 patients who underwent MRI at 1 year after particle RT, 21 (91.3%) experienced complete remission of the primary tumor. The details of the treatment response are shown in Table 2. The 3-year and 4-year LC, RC, and DMC rates were 92.3% (95% CI 82.6–100%), 84.6% (95% CI 69.0–100%), 95.8% (95% CI 88.2–100%), 87.8% (95% CI 72.7–100%), 90.2% (95% CI 78.2–100.0%) and 71.3% (95% CI 52.6–96.6%), respectively (Fig. 1B–D).

**Table 2:** Response to particle beam radiation therapy*

Response	At completion of RT	3 months after RT	6 months after RT	9 months after RT	12 months after RT
Total patients^†^	26	18	23	23	23
CR	3 (11.5%)	11(61.1%)	19(82.6%)	20(87.0%)	21(91.3%)
PR	10 (38.5%)	6(33.3%)	4 (17.4%)	2 (8.7%)	1(4.3%)
SD	13 (50.0%)	1 (5.6%)	0	0	0
PD	0	0	0	1 (4.3%)	1 (4.3%)

*Abbreviation* CR = complete response; PR = partial response; SD = stable disease

†Number of patients with information on treatment response at corresponding time points

### Acute and late toxicity

Tables 3 and 4 detail the acute and late toxicity induced by PBRT. Only two patients developed grade 3 mucositis during RT, but most of the patients (88.5%) experienced grade 1–2 toxicities, including dermatitis (24 patients, 92.3%), xerostomia (21 patients, 80.8%), hearing decrease (2 patients, 7.7%), and tinnitus (2 patients, 7.7%). Grade 1–2 late toxicities included xerostomia (16 patients, 61.6%), hearing decrease (9 patients, 34.6%), tinnitus (7 patients, 26.9%), and temporal lobe necrosis (2 patients, 7.7%). Five patients (19.2%) experienced cranial neuropathy, all of which were classified as Grade 1. Among them, two patients developed ipsilateral facial numbness and tongue deviation at 2.8 years and 3 years after the completion of treatment, respectively. The remaining three patients had cranial nerve palsy before treatment (two with mild ipsilateral facial sensory abnormalities and one with mild ipsilateral ptosis). Approximately 1.5 years after the completion of treatment, the symptoms of cranial nerve palsy worsened but remained at Grade 1. The visual acuity decreased in two of the five patients who had orbit or orbital apex invasion. Eventually, one of the patients suffered a grade 2 decrease, and the other suffered a grade 4 decrease at 1.5 and 2.5 years after the completion of radiotherapy. No late grade 3 or 5 toxicity was observed.

**Table 3:** Acute toxicities induced by particle radiation therapy

Toxicities	Grade 1	Grade 2	Grade 3
Mucositis	10 (38.5%)	13 (50.0%)	2 (7.7%)
Dermatitis	22 (84.6%)	2 (7.7%)	0
Xerostomia	19 (73.1%)	2 (7.7%)	0
Hearing impairment	2 (7.7%)	0	0
Tinnitus	2 (7.7%)	0	0

**Table 4:** Late toxicities induced by particle radiation therapy

Toxicities	Grade 1	Grade 2	Grade 4
Xerostomia	10 (38.5%)	6 (23.1%)	0
Hearing impairment	6 (23.1%)	3 (11.5%)	0
Tinnitus	7 (26.9%)	0	0
Visual acuity decreased	0	1 (3.8%)	1 (3.8%)
Cranial neuropathy	5 (19.2%)	0	0
Temporal lobe necrosis	2 (7.7%)	0	0

## Discussion

In this study, we analyzed the clinical data of 26 patients with NACC who were treated with PBRT alone. In this cohort, 92.3% of the patients had T3 or T4 disease (80.77% had T4 disease). Therefore, this study reported the survival outcomes of patients with NACC with unfavorable prognostic characteristics. The median follow-up time was 46.95 (range 17.4–77.6) months, and the 3- and 4-year OS, LC, RC, and DMC rates were 100% and 91.7%, 92.3% and 84.6%, 95.8% and 87.8%, and 90.2% and 71.3%, respectively. Although the remission of NACC was slow, complete remission was observed in 91.3% of the patients 1 year after particle RT. Severe acute toxicity was observed in only two patients (with grade 3 mucositis), and the overall toxicity profile was acceptable. Regarding late toxicity, one patient with orbital apex invasion suffered a grade 4 decrease in visual acuity. No late grade 3 or 5 toxicity was observed.

ACC is considered a radioresistant tumor with high local invasiveness and a high incidence of distant metastasis. NACC is a special subgroup of ACC that is associated with poor outcomes, with local progression and distant metastasis being the major causes of treatment failure. RT is the standard treatment for NACC due to the limited surgical access in the nasopharynx. A previous study reported the long-term outcomes of 26 patients with NACC [13]. In this study, 84.6% of the patients received photon-based RT, and nearly half of the patients (46.2%) had locally advanced disease. After a median follow-up of 48 months, the 5-year OS and locoregional control rates were 54.8% and 34.6%, respectively, and the 5-year OS for patients with stage III disease was as low as 49.4%. In another early retrospective study of 36 patients with NACC, 77.8% of whom were T3/4, photon-based RT was delivered to all patients. The median follow-up time was 61.3 months, and the 5-year locoregional failure-free survival and OS rates were 55.4% and 61.3%, respectively [14]. A recent study from Harvard Medical School included 70 patients with NACC, among whom 42% had stage 3 or 4 disease. In one study, patients with skull base invasion had poorer survival outcomes, with a 5-year OS rate of only 40%, compared to 67% for patients without skull base invasion [4].

There is increasing interest in particle RT, particularly for tumors close to critical OARs. Due to the unique physical and biological characteristics of particle beams, a higher dose can be delivered to the tumor target while sparing the surrounding OARs. Gentile et al. reported the clinical outcomes of 14 patients with locally advanced NACC who were treated with high-dose proton RT [15]. After a median follow-up of 69 months, the LC rate was 79%, and the 5-year OS rate was 59%. However, 21% of the patients experienced grade 3 or higher late toxicity, including 1 patient with grade 5 brainstem necrosis.

As heavy ion beams, carbon ion beams have a higher LET and RBE than photons and proton beams. A previous study compared the survival outcomes of 95 patients with head and neck ACC (11 patients with NACC) who were treated with IMRT alone with those who were treated with IMRT plus a carbon ion boost [16]. There were significantly better LC and OS rates in the IMRT plus CIRT group than in the IMRT alone group at 5 years (LC rate: 59.6% vs. 39.9%; OS rate: 76.5% vs. 68.7%). This may be attributable to the higher dose in the IMRT plus CIRT group than in the IMRT alone group (76.5 Gy vs. 66 Gy). Jensen et al. reported the outcomes of IMRT plus CIRT in a larger series of 309 patients with head and neck ACC [17]. After a median follow-up of 33.9 months, they reported 3- and 5-year LC rates of 83.7% and 58.5%, respectively, and 3- and 5-year OS rates of 88.9% and 74.6%, respectively. The toxicities were moderate and acceptable. A more recent study of 59 patients with NACC who were treated with IMRT plus CIRT reported that 89% of the patients had T3/4 disease [18]. With a median follow-up of 32 months, the 2- and 5-year LC rates were 83% and 49%, respectively, and the 2- and 5-year OS rates were 87% and 69%, respectively. Acute grade 3 toxicities related to IMRT plus CIRT occurred in 12% of the patients, and the incidence rate of late grade 3 toxicities was 8%. In our study, 92.3% of the patients with NACC had T3/4 disease, and the 3- and 4-year OS, LC, and RC rates were 100% and 91.7%, 92.3% and 84.6%, and 95.8% and 87.8%, respectively. All of our patients except one received proton and carbon ion mixed-beam irradiation, with the incidence of severe acute and late toxicities being 7.7% and 3.8%, respectively. In general, proton and carbon ions provide acceptable outcomes for patients with NACC.

With effective locoregional control, distant metastasis was a major treatment failure pattern in our patients, which was consistent with previous studies (4.5.18). Among the 26 patients, 10 (38.5%) developed distant metastasis, with 3- and 4-year DMC rates of 90.2% and 71.3%, respectively. It is widely known that the incidence of DM is high in patients with ACC. Unfortunately, systemic therapy has not been proven to be effective for ACC.

A few limitations to our study should be addressed. First, considering the rarity of NACC, the size of the cohort was small and the composition of the disease stages precluded prognostic analysis. Second, a longer follow-up is necessary to confirm the efficacy and safety of such treatment for NACC. Finally, only one patient experienced regional recurrence, and thus, to explore the necessity of elective neck irradiation, a phase 2 randomized trial is currently ongoing in our center.

## Conclusion

In our study, definitive PBRT provided a satisfactory 4-year OS rate for patients with locally advanced NACC. The 3- and 4-year OS, LC, RC, and DMC rates were 100% and 91.7%, 92.3% and 84.6%, 95.8% and 87.8%, and 90.2% and 71.3%, respectively. The toxicities were acceptable and mild, and severe late toxicities were observed in fewer than 5% of the patients. Long-term follow-up is necessary to confirm the efficacy and safety of definitive PBRT for patients with NACC.

## Data Availability

The clinical data analyzed during this study are available from the corresponding author on reasonable request.
